# Supplementary material for: Interplay between chromosomal alterations and gene mutations shapes the evolutionary trajectory of clonal hematopoiesis
Source: Nat Commun. 2021 Jan 12;12:338. doi: 10.1038/s41467-020-20565-7 (PMC7804935; doi:10.1038/s41467-020-20565-7)
Supplement: Supplementary file 1 — Supplementary Information [file 41467_2020_20565_MOESM1_ESM.pdf]

Supplementary Information

Interplay between chromosomal alterations and gene mutations shapes the evolutionary trajectory of clonal hematopoiesis

Gao et al.

This document contains:

Supplementary Tables 1-2

Supplementary Figures 1-21

Supplementary Methods

Supplementary References

Study	Year	Genotyping Platform	Mutation Type	Sample Size	Cohort Type	Haplotype Info
Laurie et al.	2012	SNP-array	mCA	50,222	Normal	No
Jacobs et al.	2012	SNP-array	mCA	57,853	Cancer/Normal	No
Jaiswal et al.	2014	WES	GM	17,182	Normal	-
Genovese et al.	2014	WES	GM	12,380	Normal	-
Machiela et al. (TSGII)	2015	SNP-array	mCA	24,849	Cancer/Normal	No
Zink et al.	2017	WGS	GM	11,262	Normal	-
Loh et al.	2018	SNP-array	mCA	151,202	Normal	Yes
Terao et al.	2020	SNP-array	mCA	179,417	Normal	Yes
Loh et al.	2020	SNP-array/WES(partial)	mCA/GM(3 genes)	482,789/49,960	Normal	Yes
This study	2020	Targeted Sequencing	mCA/GM(468 genes)	32,442	Cancer	No

Supplementary Table 1: Comparison with prior studies. mCA, mosaic chromosomal alterations. GM, gene mutations.

Characteristics		mCA-	mCA+	Overall
Total subjects		32096	346	32442
Age	Mean (SD)	60.5 (13.7)	69.5 (12.1)	60.6 (13.7)
Gender	Female	17821	181	
	Male	14275	165	
Race	Asian	2286	13	
	Black	2088	15	
	Other	2738	18	
	White	24984	300	
Smoking history	No	14972	131	
	Yes	15560	207	
	Missing	1564	8	
Months followup	Mean (SD)	19.7 (16.4)	18.9 (14.9)	19.7 (16.3)
Neutrophil		5.2 (3.4)	5.3 (3.1)	5.2 (3.4)
Lymphocyte		1.5 (0.7)	1.6 (1.4)	1.5 (0.7)
Monocyte		0.6 (0.3)	0.7 (0.4)	0.6 (0.3)
Hemoglobin		12.5 (1.9)	12.4 (1.8)	12.5 (1.9)
Mean corpuscular volume		89.8 (6.5)	91.5 (6.6)	89.9 (6.5)
Red cell distribution width		14.3 (2.2)	14.5 (2.1)	14.3 (2.2)
Platelets		257 (101.2)	240.2 (104.8)	256.9 (101.3)
White blood cell		7.4 (3.7)	7.8 (3.5)	7.4 (3.7)
Red blood cell		4.2 (0.6)	4.2 (0.6)	4.2 (0.6)

Supplementary Table 2: Clinical characteristics of solid tumor patients assessed for CH. Blood count parameters are reported in standard units: Neutrophil, 10^9 cells/L; Lymphocyte, 10^9 cells/L; Monocyte, 10^9 cells/L; Hemoglobin, g/dL; Mean corpuscular volume, fL; Red cell distribution width, %; Platelets, 10^9 cells/L; White blood cell, 10^9 cells/L; Red blood cell, 10^9 cells/L.

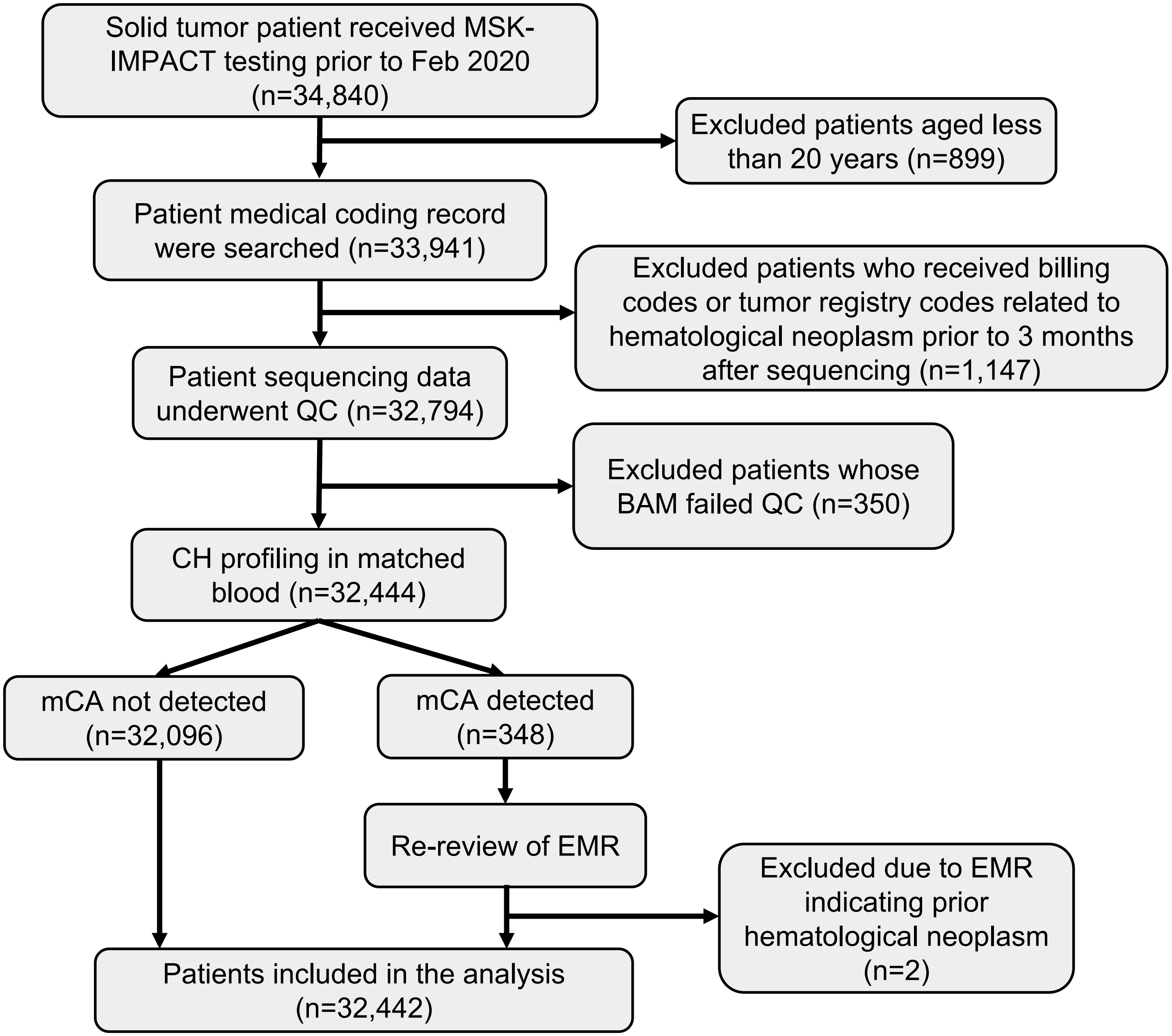

Supplementary Figure 1: CONSORT diagram for cohort selection. EMR, electronic medical records. QC, quality control. For more details please refer to the methods section.

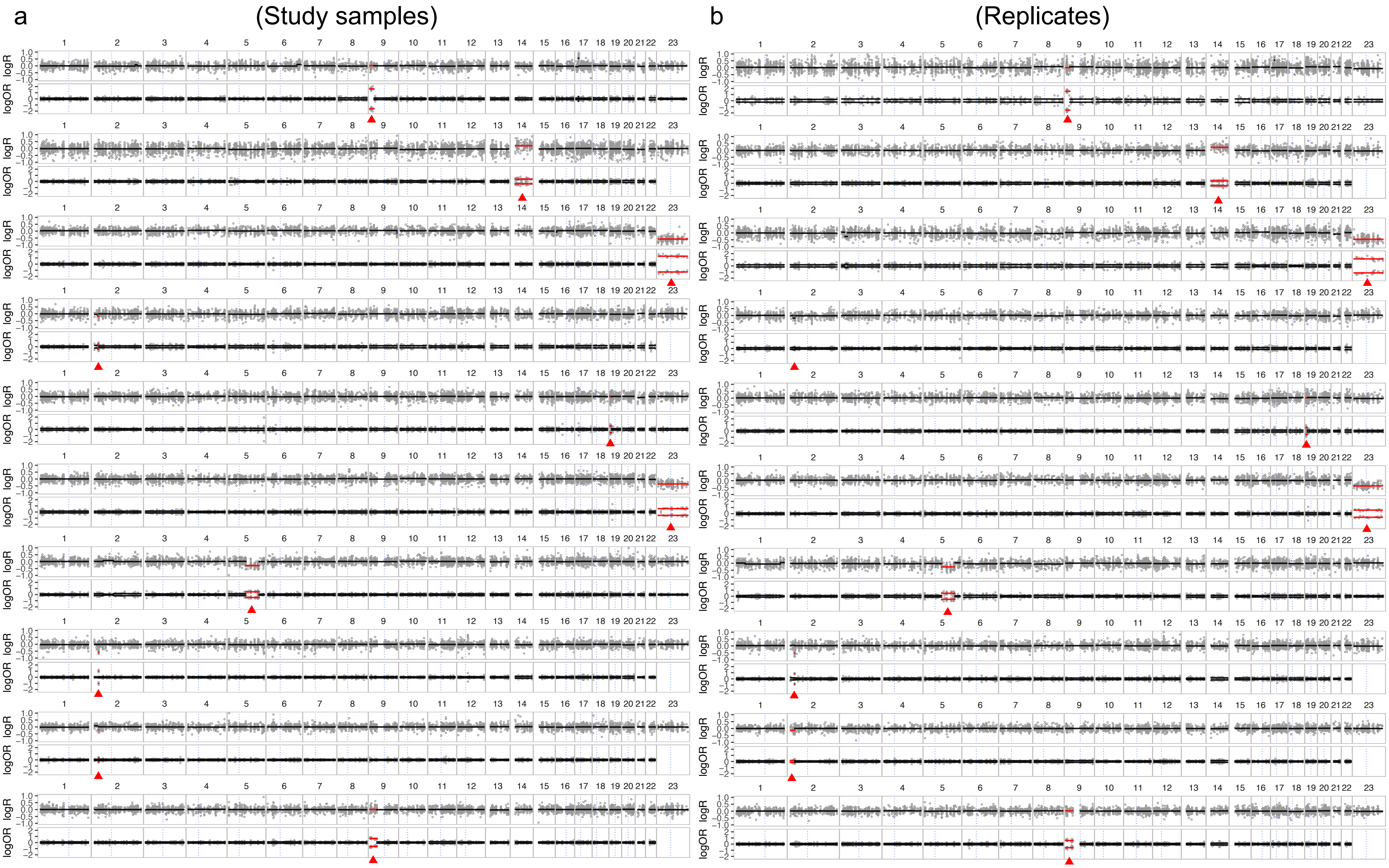

Supplementary Figure 2: mCA events are reliably reproduced in technical replicates. (A) Signal profiles of 10 study samples in the replicate cohort (n=919) with mCA events detected. (B) Signal profiles of the corresponding technical replicates (same blood sample genotyped in a separate sequencing experiment). Candidate aberrant segments are highlighted in red. Black lines indicate segment mean. Horizontal axis indicate genomic location. logR, log ratio of the coverage depth between analyzed sample and normal comparison. logOR, log allelic ratio between major and minor alleles in heterozygous SNP loci. The 4th sample had a 2p23 focal deletion that was visible but did not reach calling threshold in the replicate sample.

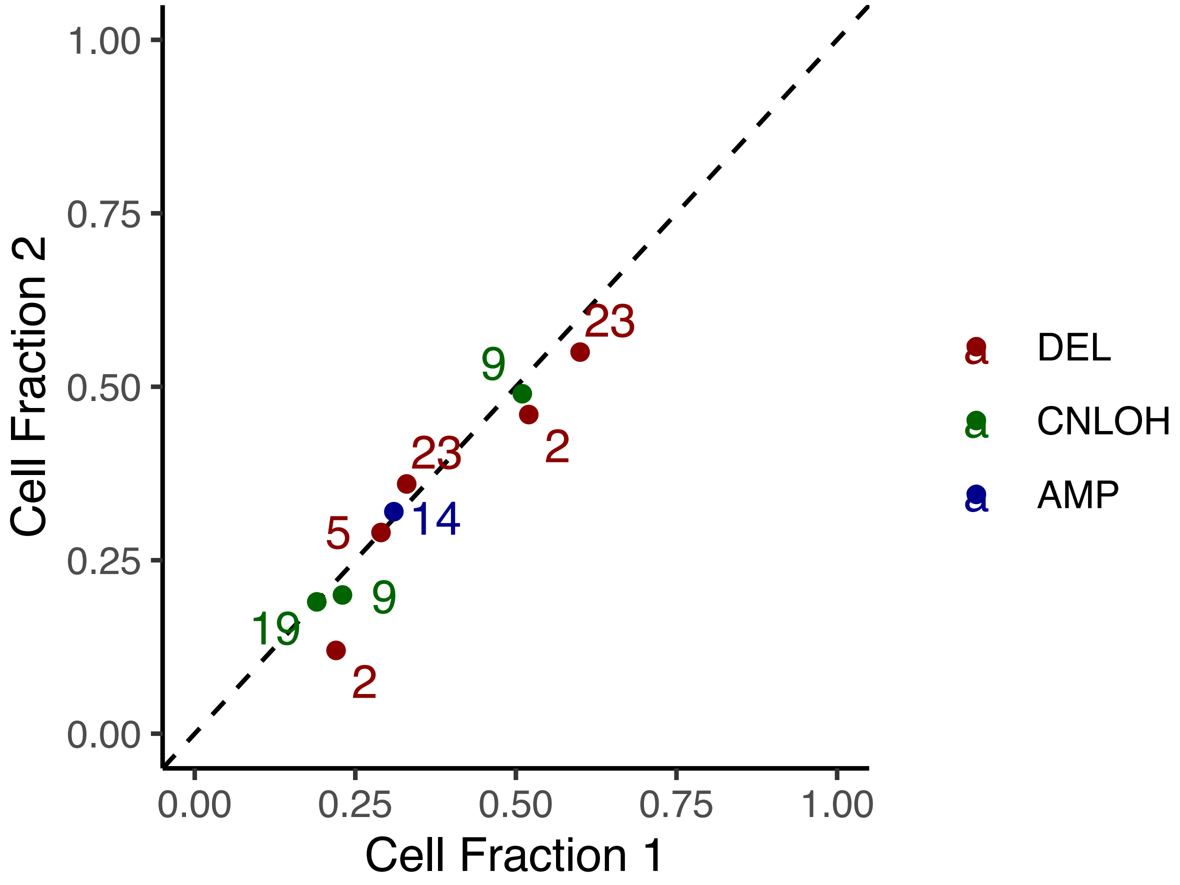

Supplementary Figure 3: Estimated cell fractions of mCAs detected in replicate samples. Cell fraction 1, estimated cell fraction in study samples. Cell fraction 2, estimated cell fraction in replicate samples. Text labels indicate chromosome number. Colors indicate alteration type (AMP, amplification; CNLOH, copy-neutral loss of heterozygosity; DEL, deletion). Diagonal line indicates equal estimated cell fractions in both samples.

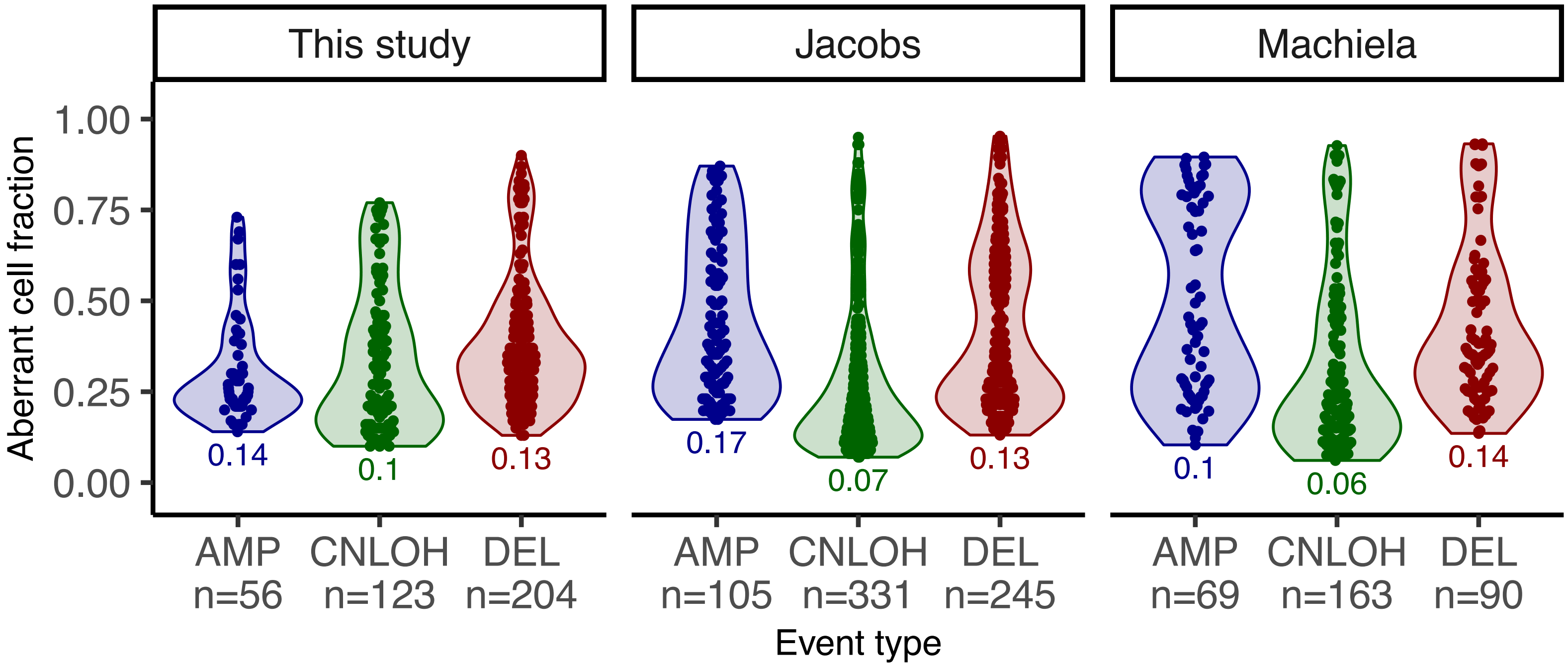

Supplementary Figure 4: Distribution of estimated cell fractions in this study and in previous studies. We compare the estimated cell fractions of detected mCA events in this study (32,442 patients) with two previous studies (Jacobs et al., 57,853 patients; Machiela et al. TSGII, 24,849 patients) based on high-density SNP arrays without haplotype phasing. Cell fractions are stratified by alteration type (AMP, amplification; CNLOH, copy-neutral loss of heterozygosity; DEL, deletion). Numbers on the bottom of violins indicate the smallest detected aberrant cell fraction. We did not include Laurie et al. in this comparison since no estimates of cell fraction was available.

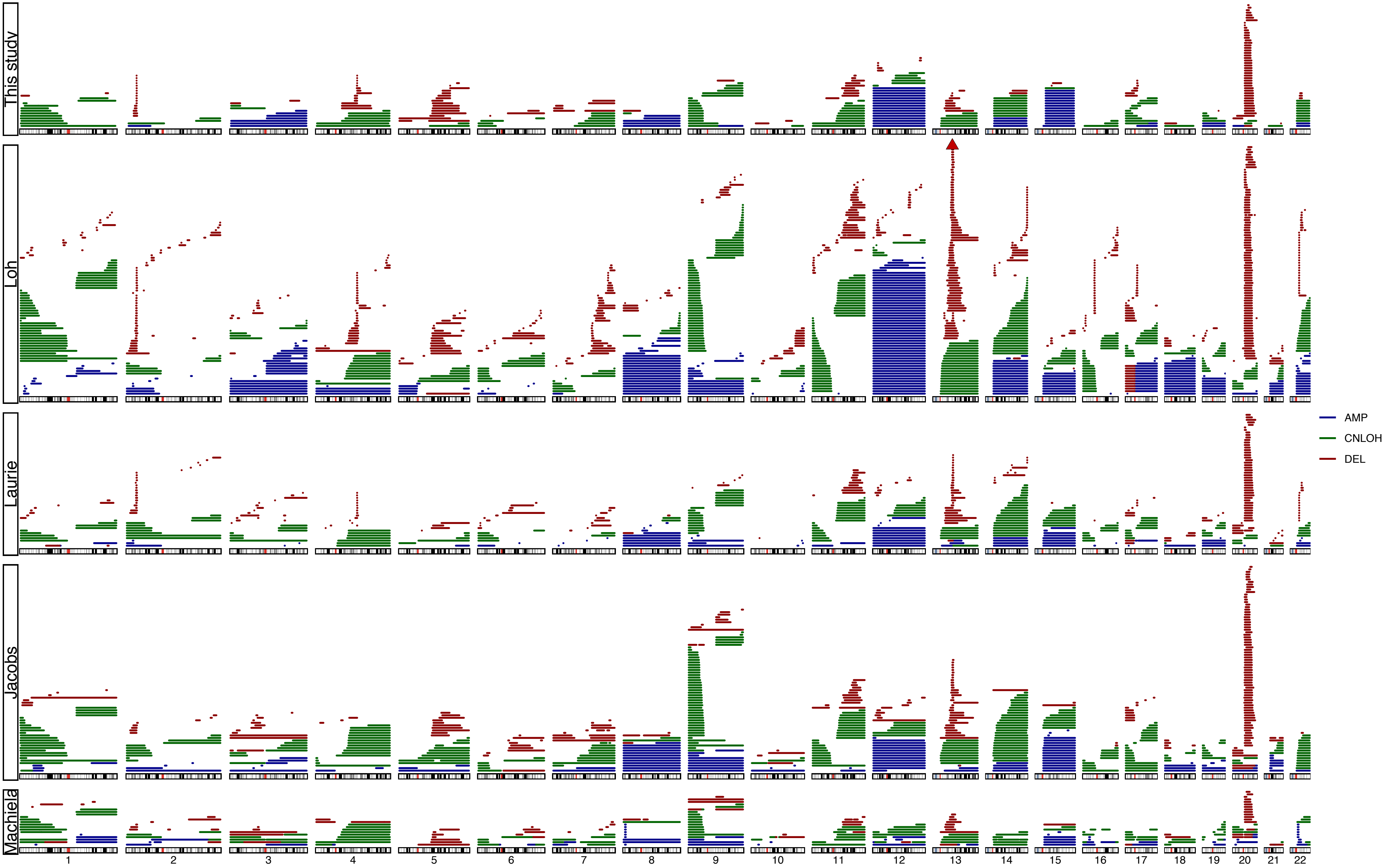

Supplementary Figure 5: Genome-wide distributions of detected mCA events in this study and in previous studies. We compare the genomic distributions of detected mCA events in this study and in previous studies (Loh et al. 2018, Laurie et al., Jacobs et al., Machiela et al.). We restricted this comparison to events that have a estimated cell fraction ≥ 10% where such estimate is available. Events are colored by alteration type (AMP, amplification; CNLOH, copy-neutral loss of heterozygosity; DEL, deletion). For display purposes, we only showed a maximum of 100 events (red triangle in Loh et al.). The event boundaries in our study might be imprecise because of unevenly spaced probe tiling.

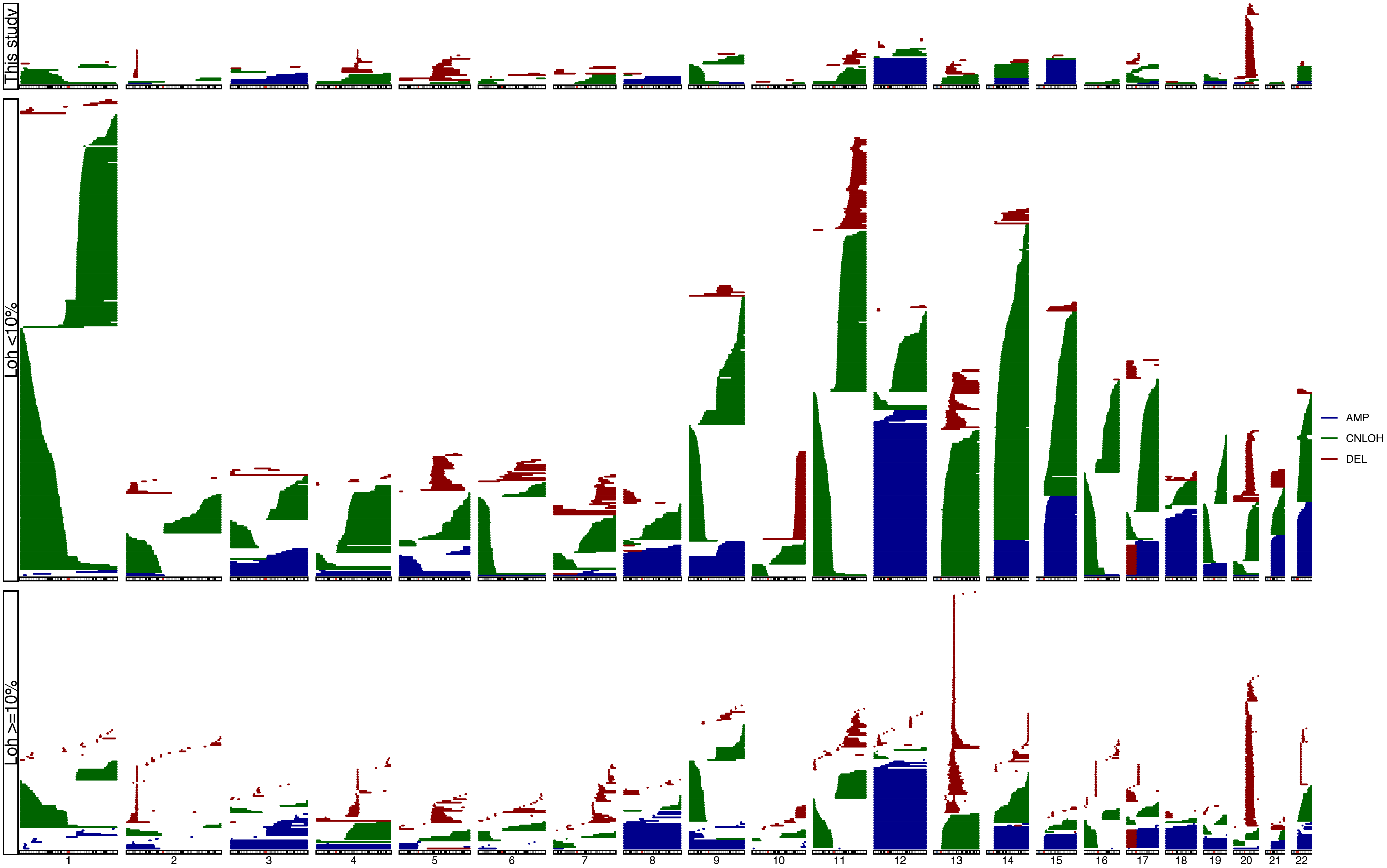

Supplementary Figure 6: Genome-wide distributions of detected mCA events with different cell fractions. We compare the genomic distributions of mCAs detected at <10% cell fraction and >=10% cell fraction in Loh et al. 2018 and in this study. Events are colored by alteration type (AMP, amplification; CNLOH, copy-neutral loss of heterozygosity; DEL, deletion).

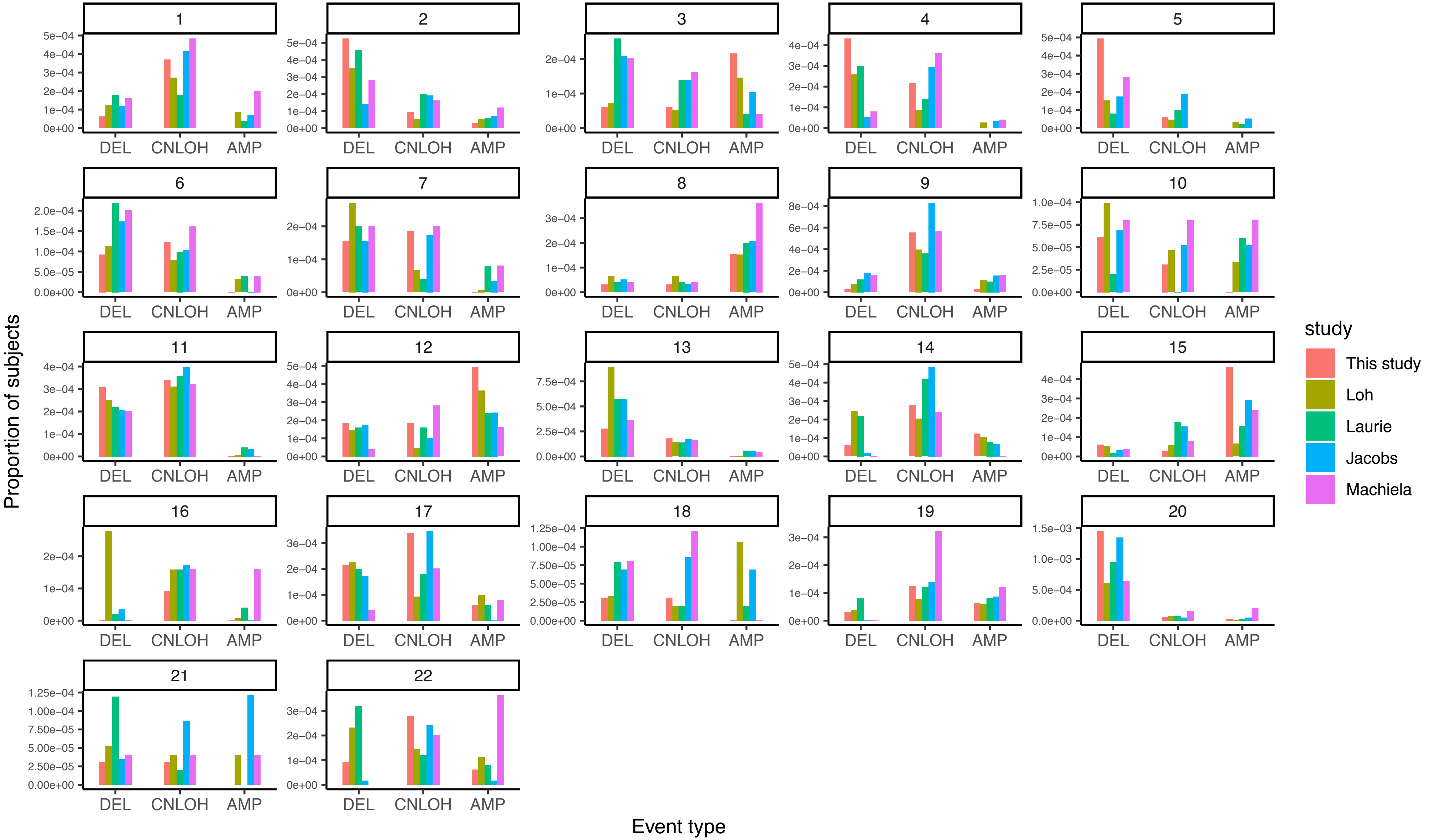

Supplementary Figure 7: Incidence of detected mCA events in this study and in previous studies. We compare the incidence (proportion of study subjects) of detected mCA events in this study and in previous studies (Loh et al. 2018, Laurie et al., Jacobs et al., Machiela et al.). We restricted this comparison to events that have a estimated cell fraction ≥ 10% where such estimate is available. Incidences are stratified by alteration type (AMP, amplification; CNLOH, copy-neutral loss of heterozygosity; DEL, deletion) and chromosome. The incidences of mCA events in our study are broadly consistent with previous work. Differences can be explained by cohort age, sex, race distribution as well as detection sensitivity. Source data are provided as a Source Data file.

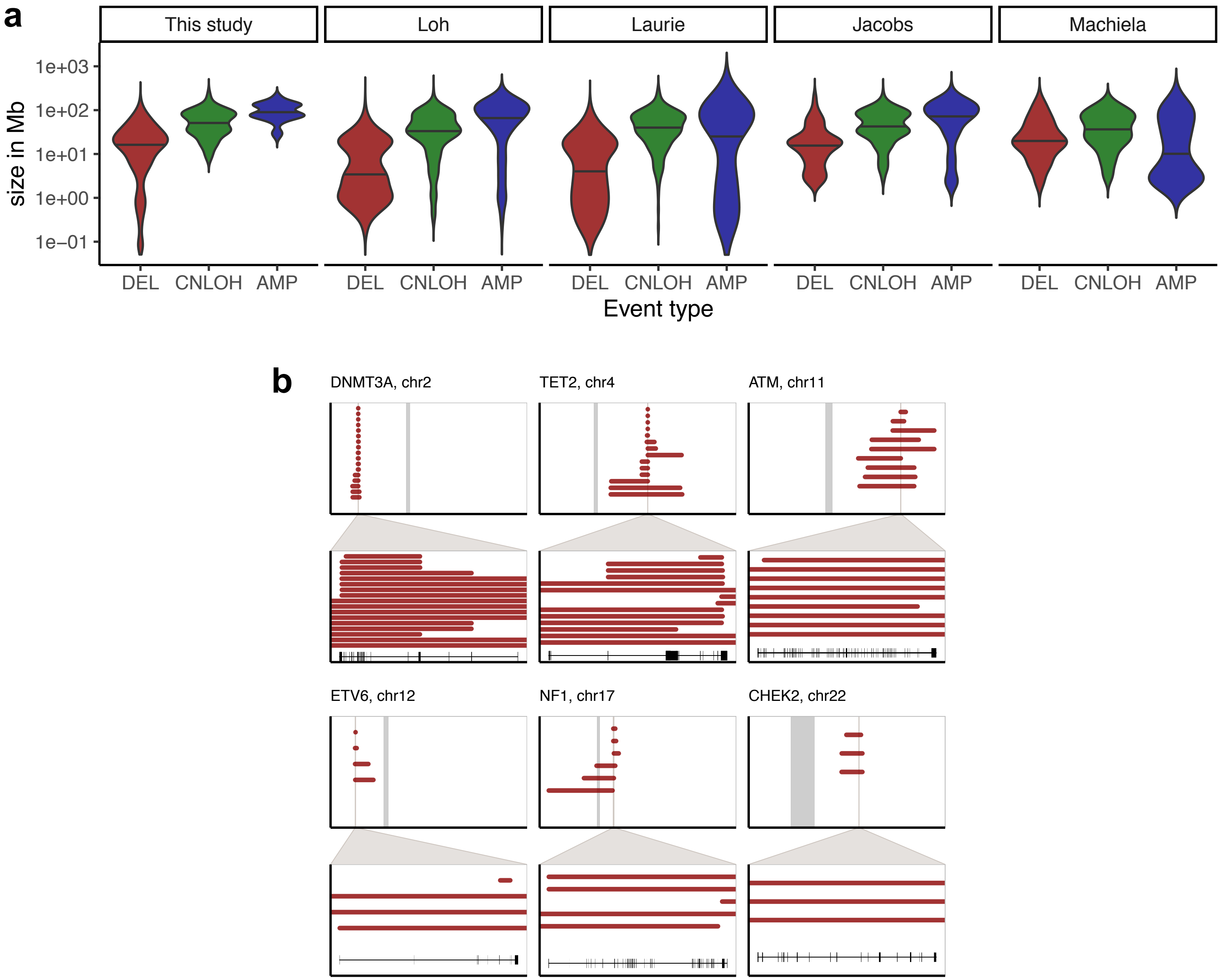

Supplementary Figure 8: Size distributions detected mCAs. (a) Sizes of detected mCA events in this study and in previous studies. We compare the sizes of detected mCA events in this study and in previous studies (Loh et al. 2018, Laurie et al., Jacbos et al., Machiela et al.). We restricted this comparison to events that have a estimated cell fraction ≥ 10% where such estimate is available. Incidences are stratified by alteration type (AMP, amplification; CNLOH, copy-neutral loss of heterozygosity; DEL, deletion). **(b)** Focal deletions detected in this study. Zoom windows expand on putative gene loci targeted by the deletions. Gene structure is indicated below the expanded view. Centromere locations are marked in gray. The event boundaries in our study might be imprecise because of unevenly spaced probe tiling.

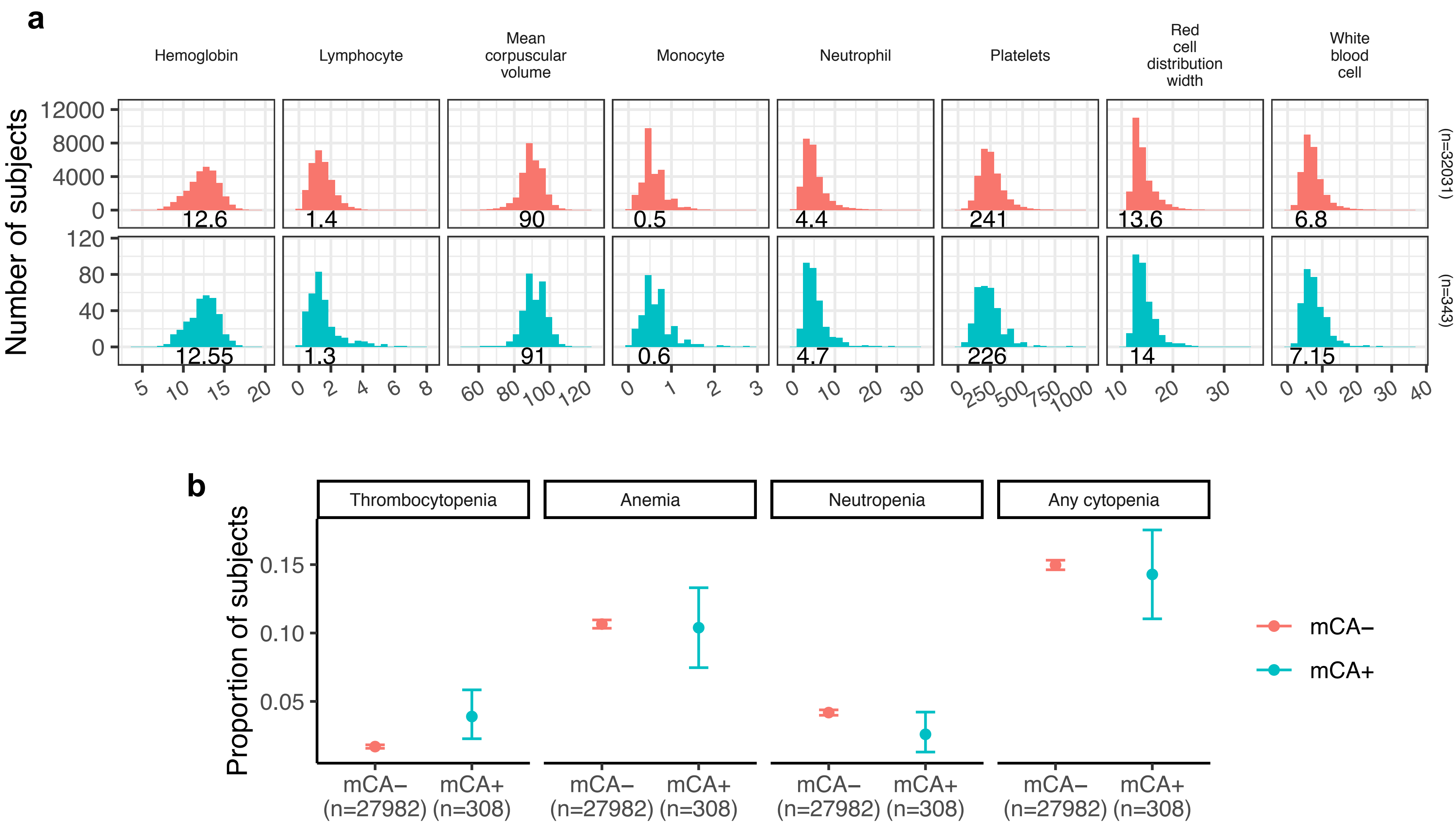

Supplementary Figure 9: Blood count distributions in patients with and without detectable mCA. (a) Distribution of patient blood counts by mCA status. Numbers below histograms denote the count median. Outliers are removed for ease of display. Overall, subjects with mCA showed a similar blood count distribution as ones without. **(b)** Incidence of cytopenias by mCA status. Observed proportion of subjects with cytopenia (shown in solid dots) are presented with 95% confidence interval (vertical bars) generated from a binomial distribution. Statuses of cytopenia were determined using the lab test closest to the timepoint of CH assessment according to the WHO criteria (anemia: hemoglobin < 10g/dL, thrombocytopenia: platelets $<100 \times 10^9$/L, neutropenia: absolute neutrophil count $<1.8 \times 10^9$/L). Overall, subjects with mCA showed a similar incidence of cytopenias as ones without. We only retained individuals with complete blood count data for all entry for the evaluation of cytopenias, hence the smaller number of individuals.

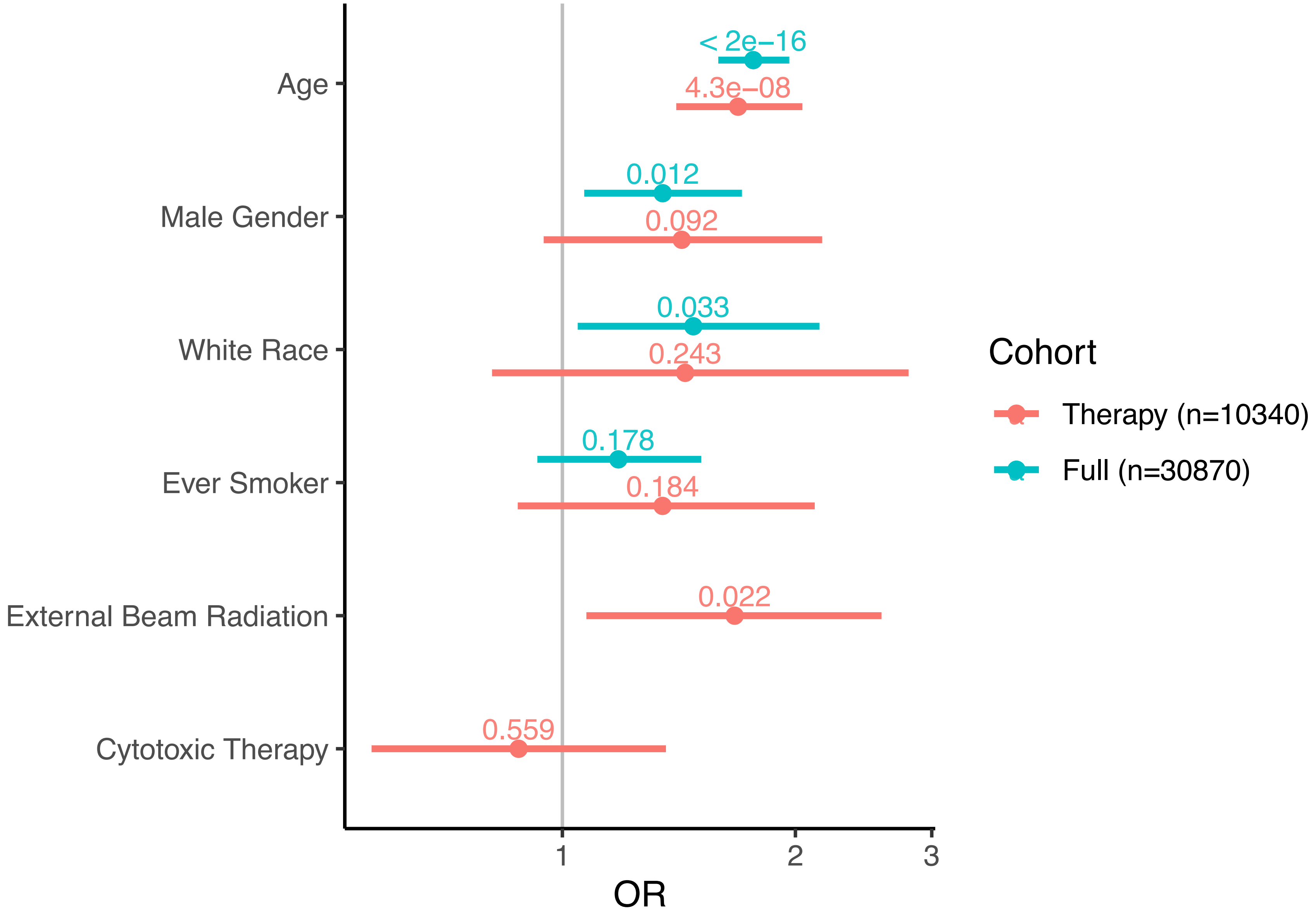

Supplementary Figure 10: Association of mCA with clinical variables. Odds ratios (solid dots), 95% confidence intervals (horizontal bars) and unadjusted *P* values (above horizontal bars) are derived from a multivariate logistic regression model. Colored in blue are the effect estimates from the full cohort with complete demographics data. Colored in red are the effect estimates from a subset of the patients with full treatment history available (therapy cohort). Only patients with complete data are included. The associations presented in this figure do not represent causal relationships. Source data are provided as a Source Data file.

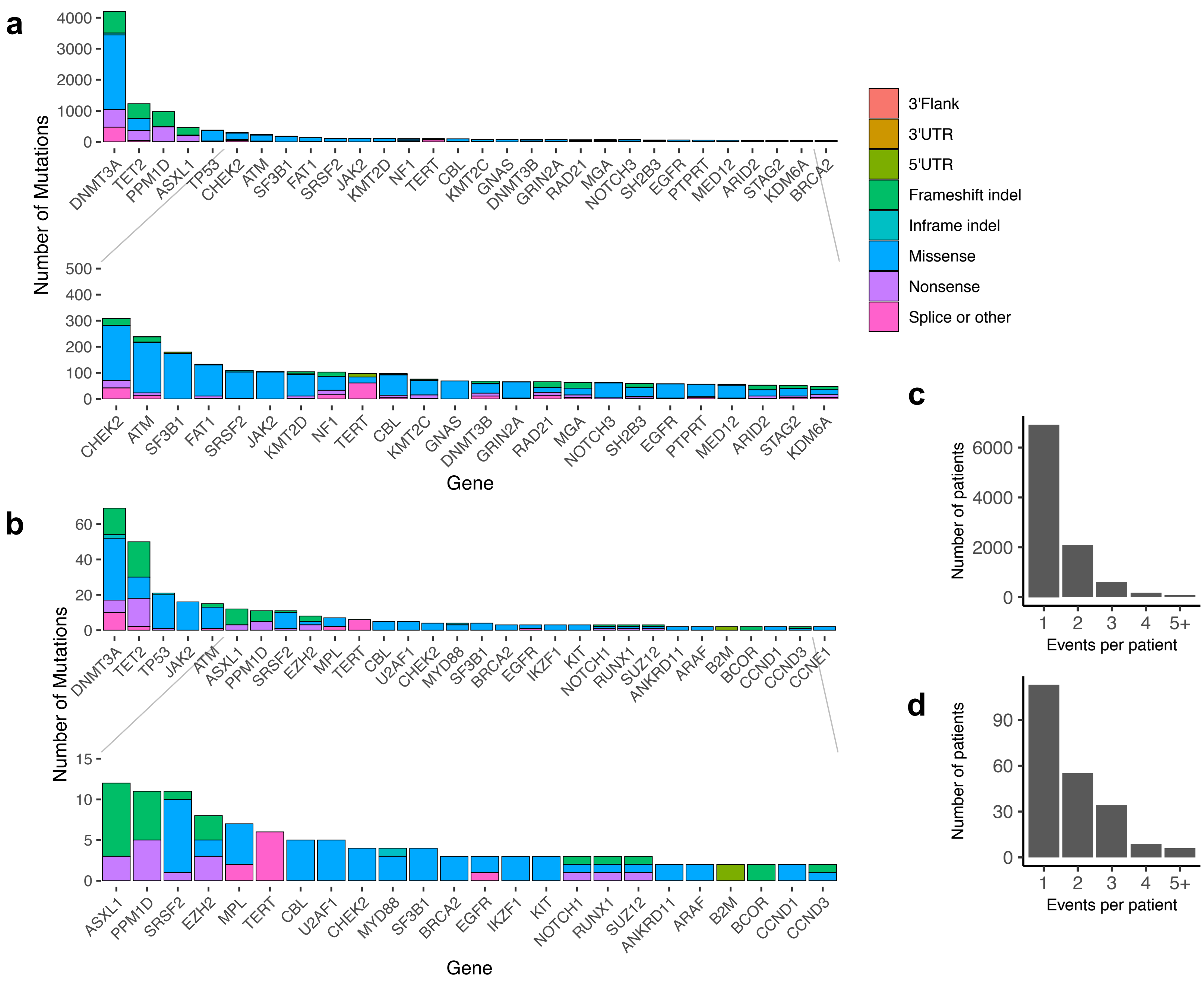

Supplementary Figure 11: Characteristics of gene mutations. (a) Top gene mutations in the entire cohort regardless of mCA status. **(b)** Top gene mutations in subset of patients with mCA. Counts are colored by variant classes. Silent mutations were not included. **(c)** Number of mutations per patient in the entire cohort overall **(d)** Number of mutations per patient in subset of patients with mCA

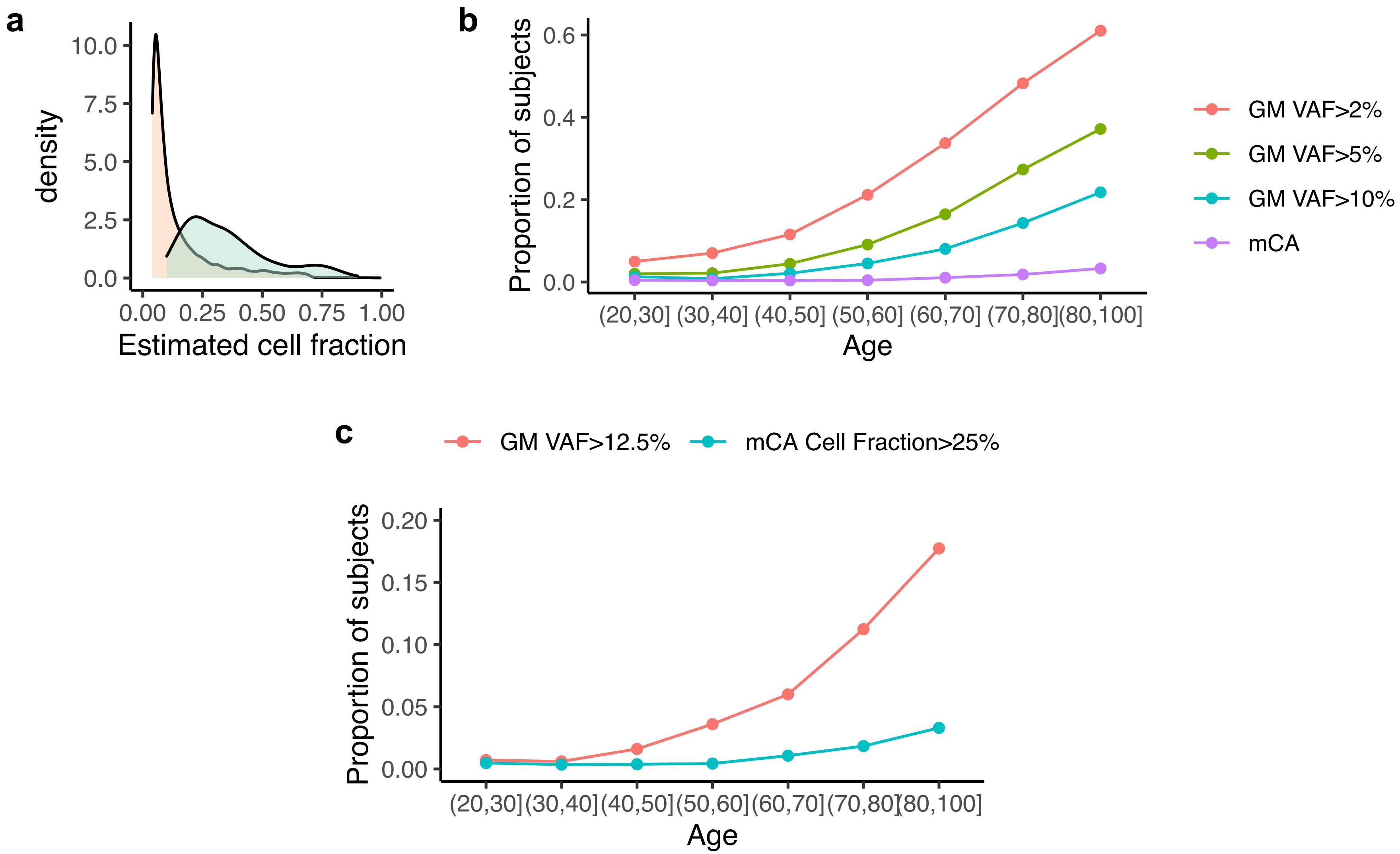

Supplementary Figure 12: Characteristics of detected gene mutations in comparison to chromosomal alterations. **(a)** Distribution of estimated cell fractions of gene mutations (yellow) and mCAs (green). Estimated cell fractions of gene mutations are calculated as VAF * 2. Gene mutations in regions affected by mCAs are excluded. **(b)** Age-related incidence of gene mutations and chromosomal alterations in CH. **(c)** Comparison of age-related incidence of gene mutations and chromosomal alterations with inferred cell fraction >25%. Estimates of mutation prevelance are dependent on the detection sensitivity and genomic coverage specific to our sequencing panel (MSK-IMPACT) as well as cohort characteristics such as gender, race, and mutagen exposures. Source data are provided as a Source Data file.

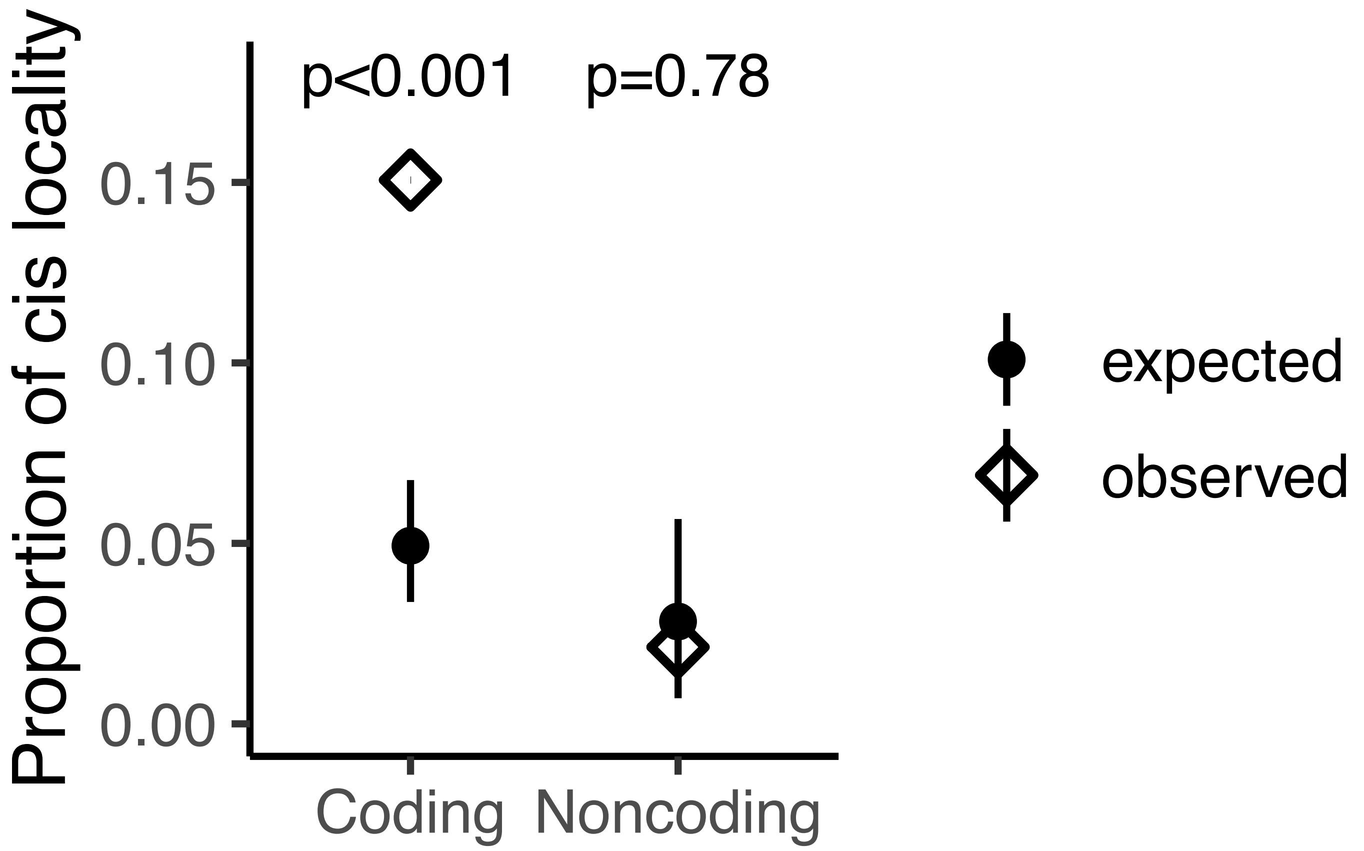

Supplementary Figure 13: Observed and expected proportion of gene mutation overlapping an mCA (in *cis*) out of all cases where they co-occurred in the same sample. Expected proportions of *cis* locality (black dots), 95% confidence intervals (vertical bars) and empirical *P* values were generated by 2,000 permutations.

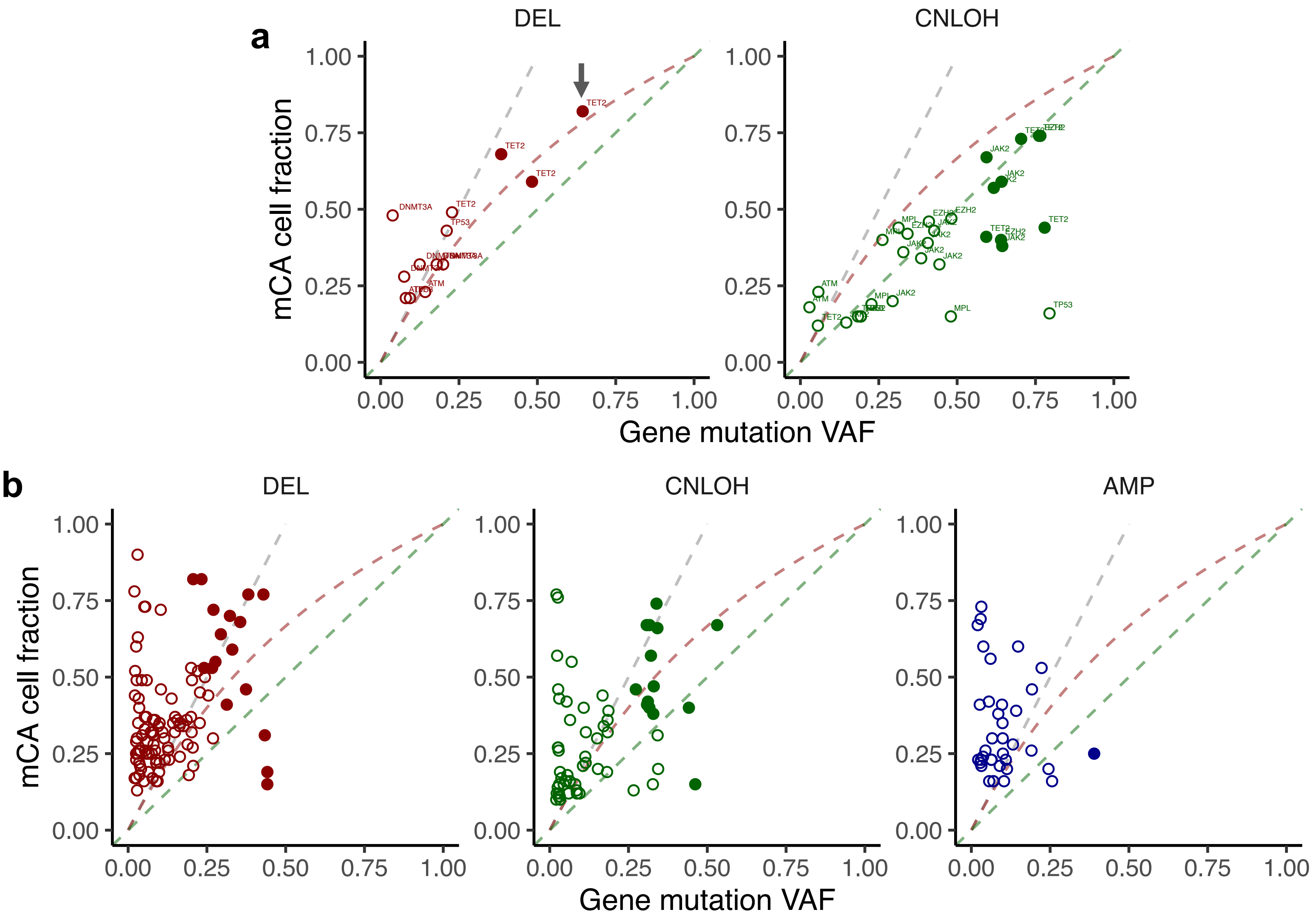

Supplementary Figure 14: VAFs and estimated cell fractions of co-occurring gene mutations and mCAs. (a) VAFs and cell fractions of gene mutations and mCAs that co-occurred in *cis*. **(b)** VAFs and cell fractions of gene mutations and mCAs that co-occurred in *trans*. For example, black arrow indicate a *TET2* mutation with VAF 64% overlapping with a deletion covering the locus at an 82% estimated cell fraction. Dashed lines indicate the expected ratios of mCA cell fraction and gene mutation VAF if they co-occur in the same clone in *cis* (red: deletion, green: CNLOH) or in *trans* (gray), provided that this clone constitutes the whole mutant cell population and that the wild type allele is deleted in cases of *cis* double hits. Solid dots represent pairs of mCAs and gene mutations that can be inferred to reside in the same clone by the pigeonhole principle (sum of inferred cell fraction > 100%). In these cases, deviations from the expected mutant fractions may indicate a mixture of clones with one or both mutations, and/or noise in measurement. The estimated cell fraction of gene mutations are calculated as 2 * VAF for those in *trans* of mCA, 1 * VAF for those in *cis* of CNLOH, and 2 * VAF/(1+VAF) for those in *cis* of deletions.

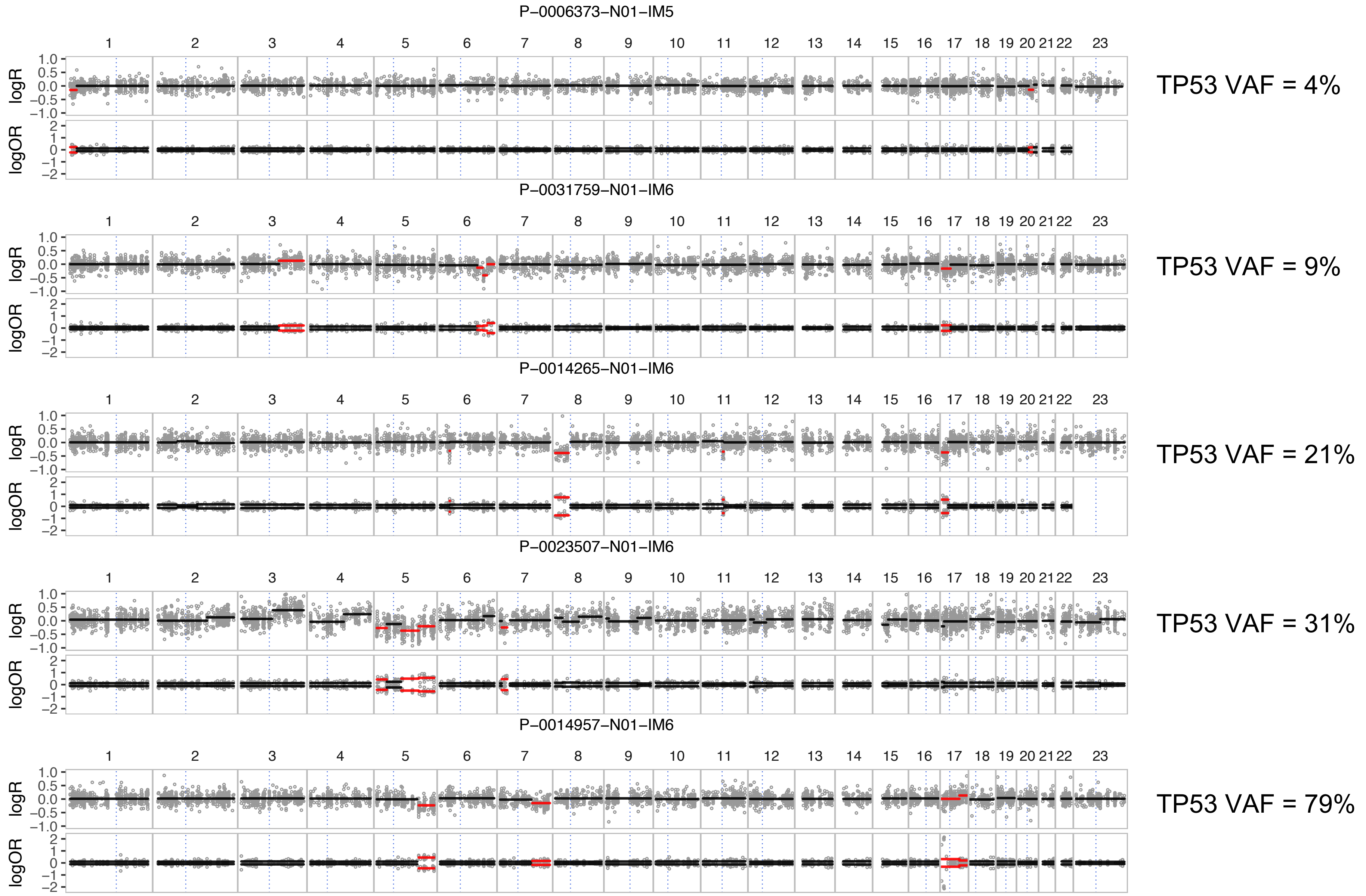

Supplementary Figure 15: Cases of multiple chromosomal aberrations with a TP53 co-mutation.
Aberrant regions detected are highlighted in red.

Comutation	Cytopenia	Lymphocytosis	Normal	Total
(chr11CNLOH | chr11DEL) & ATM	1	0	2	3
(chr4DEL | chr4CNLOH) & TET2	1	0	8	9
chr12AMP & (NOTCH1 | MYD88 | FBXW7)	0	1	5	6
chr13DEL & ATM	1	0	1	2
(chr17CNLOH | chr17DEL | chr17AMP) & TP53	1	2	1	4
chr1CNLOH & MPL	2	0	2	4
chr20DEL & (U2AF1 | TERT)	1	0	4	5
chr2DEL & DNMT3A	0	0	5	5
chr3AMP & TP53	0	0	2	2
chr4DEL & (SRSF2 | ATM | CHEK2 | ASXL1)	0	0	4	4
chr5DEL & TP53	2	0	3	5
chr7DEL & (TP53 | PPM1D)	0	0	1	1
chr7CNLOH & EZH2	1	0	5	6
chr8AMP & TET2	0	0	3	3
chr9CNLOH & JAK2	0	0	11	11
Total	10	3	57	70

Supplementary Figure 16: Blood count abnormalities in individuals with recurrent co-mutations. Statuses of cytopenia and lymphocytosis were determined using the lab test closest to the timepoint of CH assessment according to the WHO criteria (anemia: hemoglobin < 10g/dL, thrombocytopenia: platelets <100× 10^9/L, neutropenia: absolute neutrophil count <1.8× 10^9/L, lymphocytosis: absolute lymphocyte count > 5 x 10^9/L). Under co-mutation category, "|" indicates OR relationship, "&" indicates AND relationship between the mutations.

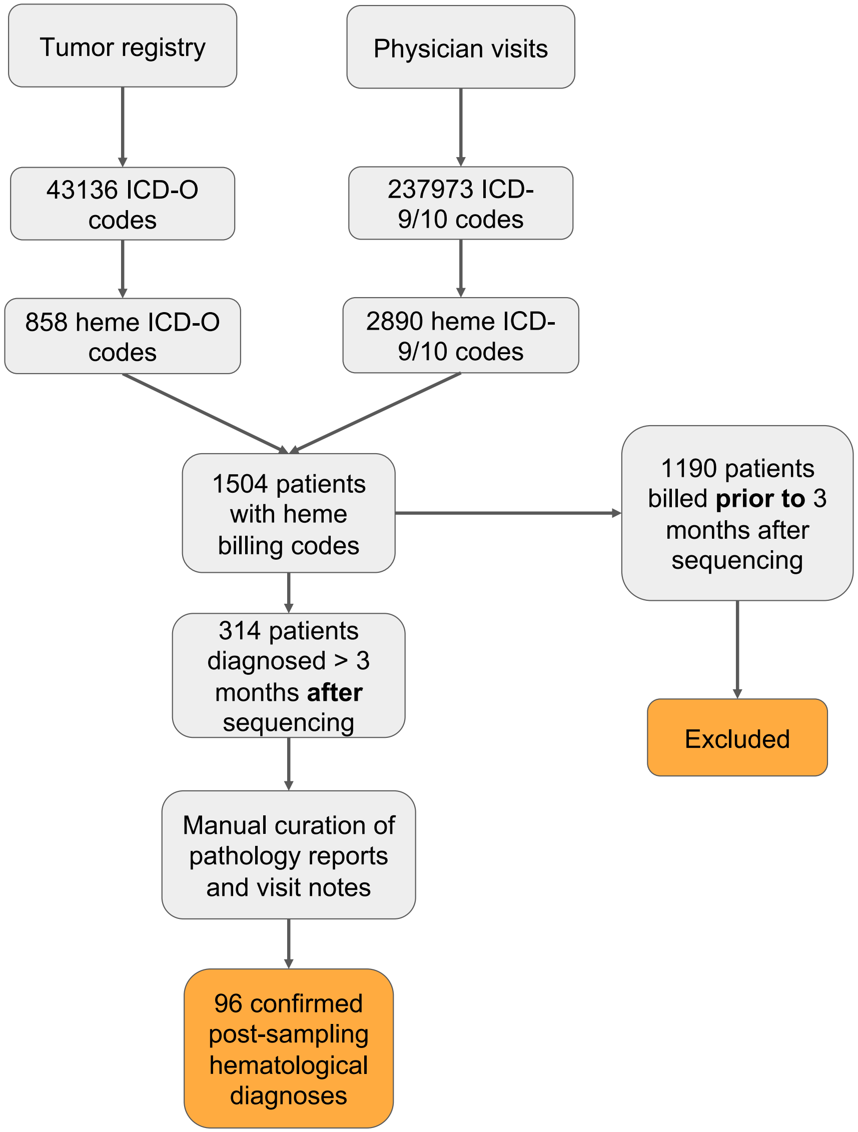

Supplementary Figure 17: Identification of hematological diagnosis using medical codes and clinical records.

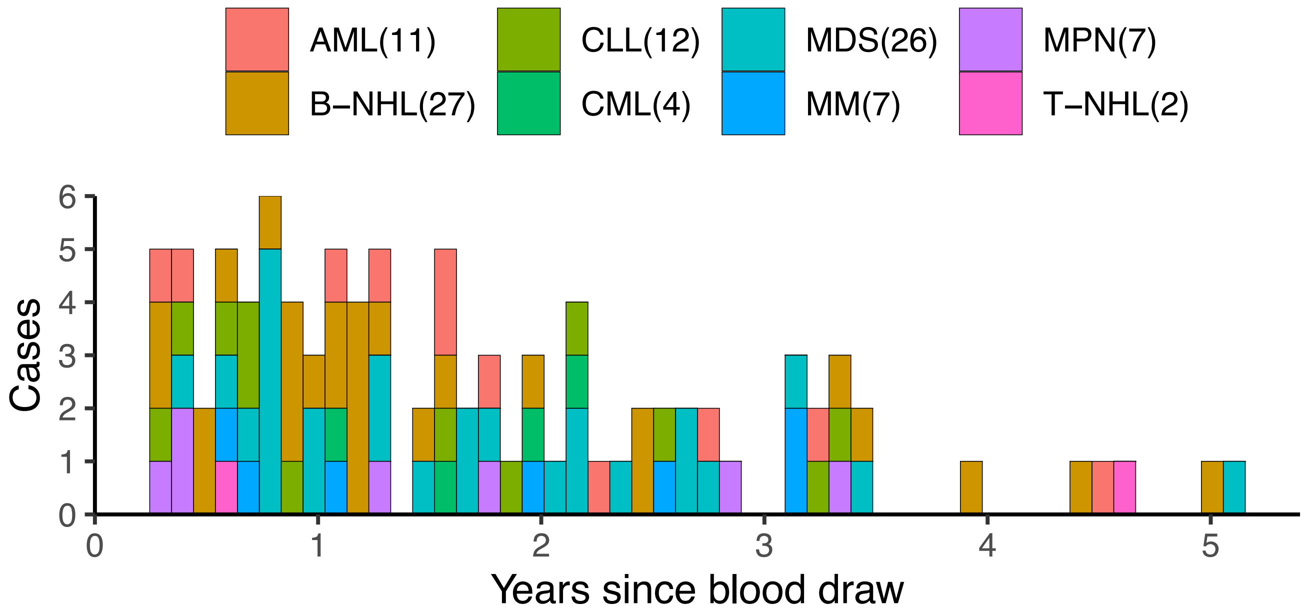

Supplementary Figure 18: Hematological cancer diagnoses >3 months after blood draw.

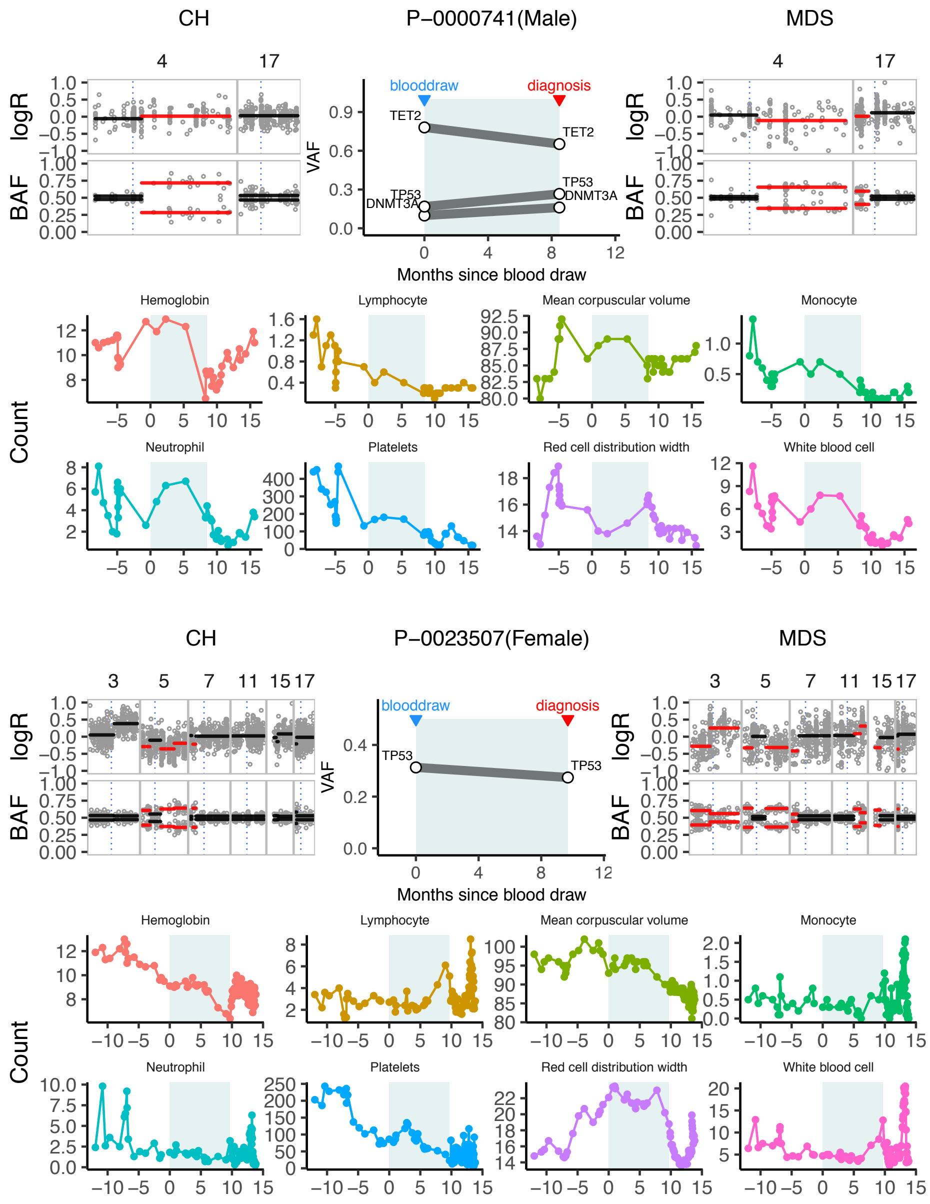

Supplementary Figure 19: **Clonal evolution involving mCA in two additional patients.** Both were solid tumor patients who developed myeloid neoplasm following initial blood collection.

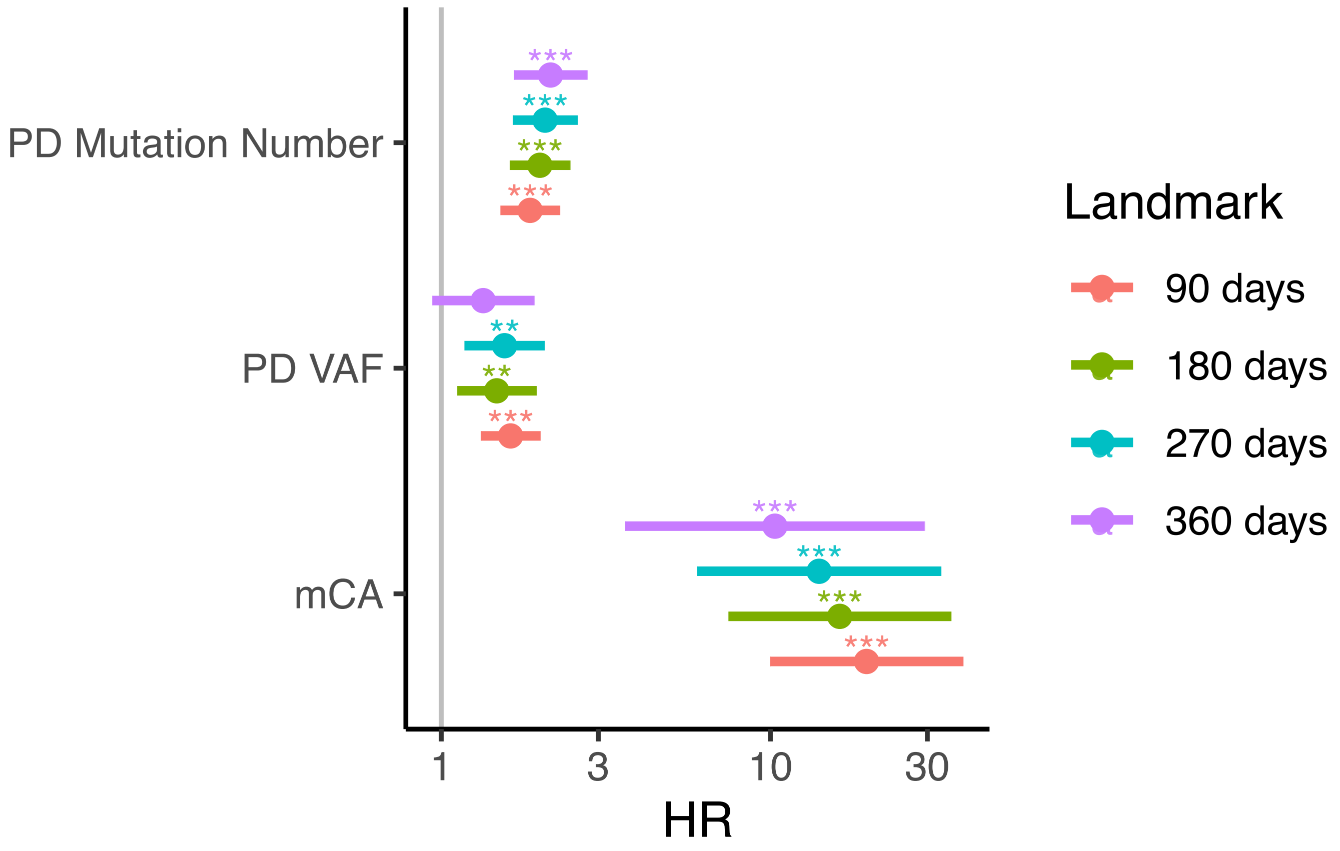

Supplementary Figure 20: CH features and risk of subsequent leukemia. Hazard ratios (solid dots) and 95% confidence intervals (horizontal bars) are derived from a multivariable cause-specific Cox regression model with different landmarks since initial blood draw (days). Significance of unadjusted *P* values are shown above the horizontal bar (*$P \leq 0.05$, **$P \leq 0.01$, ***$P \leq 0.001$). The exact *P* values can be found in Source Data. We defined events of interest as any leukemia (MDS, MPN, AML, CML, CLL). All models were adjusted for patient age. For PD VAF, HR of 10% increment were shown. PD mutation number was included as an integer variable, while mCA as a binary indicator. With each chosen landmark, all patients who either received a diagnosis or died before the landmark were excluded from the analysis, and the start of follow up was set at the landmark. Source data are provided as a Source Data file.

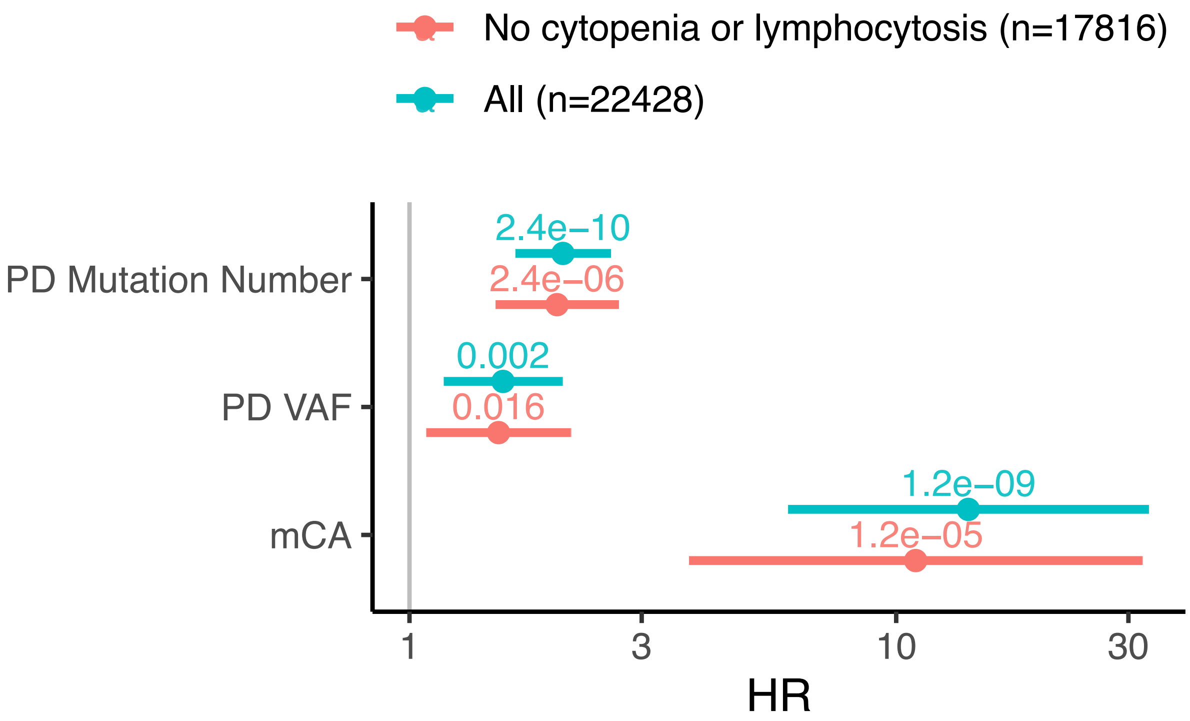

Supplementary Figure 21: Hazard ratios of CH features in individuals with and without blood abnormalities. Hazard ratios (solid dots), 95% confidence intervals (horizontal bars) and unadjusted *P* values (above horizontal bars) are derived from a multivariable cause-specific Cox regression model with a 9 month landmark. The HR for 10% increment in putative driver (PD) VAF was shown. mCA status was included as a binary indicator. Statuses of cytopenia and lymphocytosis were determined using the lab test closest to the timepoint of CH assessment according to the WHO criteria (anemia: hemoglobin < 10g/dL, thrombocytopenia: platelets <100×10^9/L, neutropenia: absolute neutrophil count <1.8×10^9/L, lymphocytosis: absolute lymphocyte count > 5×10^9/L). Source data are provided as a Source Data file.

Supplementary Methods

QC of mCA calls. We applied several empirical thresholds to reduce the rate of false positive calls. We required that the *P* value of the chi-squared score for an aberrant segment to be $<1^{-10}$ and the *P* value of the Z_y score to be $<10^{-3}$. The stringent *P* value cutoff reflects the large number of hypothesis testings (as many as the total number of segments explored in all individuals in the cohort) as well as our higher confidence to detect aberrant clones that exhibit larger deviations in both signal tracks. We also required the error score (Euclidean distance from the expected logR/logOR ratio given the inferred cell fraction) to be <0.06. To eliminate potential constitutional germline duplications, we filtered out any gain events with an estimated cell fraction > 80%, any gain events with logR > 0.5, or any gain events with size smaller than 24Mb (since germline duplications are characteristically short[13]). We filtered out any LOH events with size smaller than 8Mb since interstitial LOHs are rare and these calls are likely artifacts[13]. After these filters, we observed that certain genomic regions are prone to recurrent artifacts: chr6<58Mb, chr7>61Mb, and chr2>50Mb. To address this, we eliminated heterozygous SNPs with anomalous logR distributions in the PON (i.e. in the logR blacklist) in those areas at the initial signal processing step of FACETS-CH. We additionally identified regions with recurrent focal deletions that are not reported in any other CH studies or any cancer types in cBioPortal: chr2:110Mb-130Mb and chr11:60Mb-70Mb. Similarly, chr7:40Mb-70Mb (around the centromere region) showed recurrent interstitial CNLOH that were likely to be artifacts. We filtered out events from these areas.

QC of gene mutation calls. All genotypes were calculated using sequencing reads and bases with a quality value of at least 20. We removed variants that were found (with a VAF of >2% at least once) in a panel of sequencing data from 300 blood samples obtained from persons under 20 years of age and without evidence of clonal hematopoiesis. We filtered single nucleotide deletions within a homopolymer stretch of (>=3 base repetition) of the same deleted base pair, single nucleotide substitutions completing a stretch of a >= 5bp-

long homopolymer (E.g. GGCGG -> GGGGG) in-frame deletions or insertions in a highly repetitive region (DUST algorithm score of >=5), and variants with unequal proportions of forward/reverse direction supporting reads based on a fisher test. We required a variant allele fraction of at least 2% and at least 10 supporting reads. All called mutations were genotyped in the patient matched tumor sample. Because somatic mutations in the blood would be expected to be detected in the blood but not other tissue compartments, we compared the variant allele fraction (VAF) of mutations in the blood compared to the matched tumor. Variant calls that were present in the blood with a VAF of at least twice that in the tumor or 1.5 times the VAF if the tumor biopsy site was a lymph node were considered somatic. This ratio was chosen based on minimizing sensitivity and specificity of gene mutation calls through simulations of leukocyte contamination in the tumor.

Curation of hematological cancer diagnoses and ICD codes. We used medical coding data to identify patients who received a hematological malignancy diagnosis. Medical codes related to hematological malignancies are defined as any ICD-9 code in the range 200-208 or 238.4-238.7, any ICD-10 code in the range C81-C96 or D45-D47, and any ICD-O histology code present in the NIH SEER hemeDB (https://seer.cancer.gov/seertools/hemelymph/). ICD-O-2 codes were converted to ICD-O-3 according to the conversion guide provided by SEER (https://seer.cancer.gov/tools/conversion/). A date cutoff is applied to identify patients who had an active hematological diagnosis prior to or within 3 months of blood collection, whom we excluded from all analysis. We additionally reviewed the electronic medical records of mCA positive patients to ensure that these patients did not have a prior hematological malignancy before receiving care at MSK, leading to the exclusion of 2 additional patients. We used medical codes received more than 3 months after initial blood collection to identify patients who subsequently developed a hematological cancer during the followup period. Since not all medical billing codes reflect a bona fide diagnosis, we manually reviewed the physician visit notes, hematopathology reports, and molecular testing results (mainly IMPACT-HEME, a NGS targeted capture assay for hematological diseases) of these patients. The review identified 96 patients with a valid diagnosis.

Supplementary References

1. Loh, P.-R. et al. Insights into clonal haematopoiesis from 8,342 mosaic chromosomal alterations. Nature 559, 350–355 (2018).

2. Jacobs, K. B. et al. Detectable clonal mosaicism and its relationship to aging and cancer. Nat. Genet. 44, 651–658 (2012).

3. Laurie, C. C. et al. Detectable clonal mosaicism from birth to old age and its relationship to cancer. Nat. Genet. 44, 642–650 (2012).

4. Machiela, M. J. et al. Characterization of large structural genetic mosaicism in human autosomes. Am. J. Hum. Genet. 96, 487–497 (2015).

5. Terao, C. et al. Chromosomal alterations among age-related haematopoietic clones in Japan. Nature (2020) doi:10.1038/s41586-020-2426-2.

6. Loh, P.-R., Genovese, G. & McCarroll, S. A. Monogenic and polygenic inheritance become instruments for clonal selection. Nature (2020) doi:10.1038/s41586-020-2430-6

7. Zink, F. et al. Clonal hematopoiesis, with and without candidate driver mutations, is common in the elderly. Blood 130, 742–752 (2017).
