# Supplementary material for: Interplay between chromosomal alterations and gene mutations shapes the evolutionary trajectory of clonal hematopoiesis
Source: Nat Commun. 2021 Jan 12;12:338. doi: 10.1038/s41467-020-20565-7 (PMC7804935; doi:10.1038/s41467-020-20565-7)
Supplement: Supplementary file 3 — Description of Additional Supplementary Files [file 41467_2020_20565_MOESM3_ESM.pdf]

Description of Additional Supplementary Files

File Name: Supplementary Data 1
Description: CH gene mutation and mCA calls
