# Supplementary material for: Interplay between chromosomal alterations and gene mutations shapes the evolutionary trajectory of clonal hematopoiesis
Source: Nat Commun. 2021 Jan 12;12:338. doi: 10.1038/s41467-020-20565-7 (PMC7804935; doi:10.1038/s41467-020-20565-7)
Supplement: Supplementary file 5 — Reporting Summary [file 41467_2020_20565_MOESM5_ESM.pdf]

Reporting Summary

Nature Research wishes to improve the reproducibility of the work that we publish. This form provides structure for consistency and transparency in reporting. For further information on Nature Research policies, see our [Editorial Policies](#) and the [Editorial Policy Checklist](#).

Statistics

For all statistical analyses, confirm that the following items are present in the figure legend, table legend, main text, or Methods section.

n/a	Confirmed
☐	☒ The exact sample size (n) for each experimental group/condition, given as a discrete number and unit of measurement
☐	☒ A statement on whether measurements were taken from distinct samples or whether the same sample was measured repeatedly
☐	☒ The statistical test(s) used AND whether they are one- or two-sided<br><i>Only common tests should be described solely by name; describe more complex techniques in the Methods section.</i>
☐	☒ A description of all covariates tested
☐	☒ A description of any assumptions or corrections, such as tests of normality and adjustment for multiple comparisons
☐	☒ A full description of the statistical parameters including central tendency (e.g. means) or other basic estimates (e.g. regression coefficient) AND variation (e.g. standard deviation) or associated estimates of uncertainty (e.g. confidence intervals)
☐	☒ For null hypothesis testing, the test statistic (e.g. F , t , r) with confidence intervals, effect sizes, degrees of freedom and P value noted<br><i>Give P values as exact values whenever suitable.</i>
☒	☐ For Bayesian analysis, information on the choice of priors and Markov chain Monte Carlo settings
☒	☐ For hierarchical and complex designs, identification of the appropriate level for tests and full reporting of outcomes
☐	☒ Estimates of effect sizes (e.g. Cohen's d , Pearson's r), indicating how they were calculated

Our web collection on [statistics for biologists](#) contains articles on many of the points above.

Software and code

Policy information about [availability of computer code](#)

Data collection	Queries to Darwin Digital Portal to collect patient clinical data was made in python (Version 3.7.4).
Data analysis	All analysis was conducted using R (version 3.6.1) and Jupyter Lab (version 1.1.4). A custom algorithm written in R (FACETS-CH) was used to call mosaic chromosomal alterations (MCAs) from sequencing data. A github repository containing analysis notebooks and the FACETS-CH method is available for reviewers (username: reviewer.code.data@gmail.com, password: Reviewer411, or directly clone the repository by git clone https://reviewer-repo:Reviewer411@github.com/reviewer-repo/CH.git).

For manuscripts utilizing custom algorithms or software that are central to the research but not yet described in published literature, software must be made available to editors and reviewers. We strongly encourage code deposition in a community repository (e.g. GitHub). See the Nature Research [guidelines for submitting code & software](#) for further information.

Data

Policy information about [availability of data](#)

All manuscripts must include a [data availability statement](#). This statement should provide the following information, where applicable:

- Accession codes, unique identifiers, or web links for publicly available datasets
- A list of figures that have associated raw data
- A description of any restrictions on data availability

mCA and gene mutation calls are available in the Supplementary Data. Source data to reproduce figures presented in the paper are included with this paper. NIH SEER hemeDB can be accessed at <https://seer.cancer.gov/seertools/hemelymph>. MSK tumor registry and Darwin Digital Portal are for hospital internal use only and are not publicly available for patient privacy or legal reasons. The remaining data are available within the Supplementary Information or available from the authors upon request.

Field-specific reporting

Please select the one below that is the best fit for your research. If you are not sure, read the appropriate sections before making your selection.

☒ Life sciences ☐ Behavioural & social sciences ☐ Ecological, evolutionary & environmental sciences

For a reference copy of the document with all sections, see [nature.com/documents/nr-reporting-summary-flat.pdf](https://www.nature.com/documents/nr-reporting-summary-flat.pdf)

Life sciences study design

All studies must disclose on these points even when the disclosure is negative.

Sample size	Our study cohort included 32,442 solid tumor patients who underwent prospective sequencing (MSK-IMPACT) from 1/1/2014 to 2/1/2020. The appropriate sample size was determined by the population frequency of mCAs (~1%) in order to collect at least 300 representative events across the genome.
Data exclusions	Since the study focuses on non-malignant blood somatic mutations, we excluded any patient who had a hematological neoplasm diagnosis prior to or less than 3 months after IMPACT sequencing for all analysis. The 3 months cutoff after sequencing is to capture the diagnosis of patients who were undergoing workup at the time of sequencing. We excluded samples whose sequencing failed quality control metrics (criteria outlined in the methods). We excluded patients with age younger than 20 for all analysis, since their samples were used in the panel of normal as reference for calling chromosomal alterations. The above exclusion criteria were established before variant calling and data analysis. After mCA calling, we excluded 2 patients who had a prior hematological cancer diagnosis before presenting at MSK not recorded by the medical billing codes, identified through a review of all mCA positive individuals.
Replication	To assess the reproducibility of the mCA calling method, we applied the same calling procedure to 919 paired replicate samples. All data were collected from real-world patients; no experiments were performed. The replicate samples were identified retrospectively from patients who received multiple IMPACT testings for their solid tumor using the same blood sample as the normal comparison, in separate sequencing runs. No replicate samples were found for the remaining samples in the cohort. We excluded 5 replicate samples that failed sequencing quality control (criteria outlined in the methods). Out of 10 total variants that were called in the replicate cohort, all were reproduced in a separate sequencing run.
Randomization	There was no randomization or concurrent control group as there was no intervention and these data are part of an observational cohort. In the analysis to identify risk factors of mCA, we performed multivariable logistic regressions adjusted for gender, race, age and smoking status. To evaluate the association between CH features and the risk of subsequent hematological malignancies, we performed cause-specific Cox proportional hazards regression adjusted for age. Multivariable analysis was not used for the remaining parts of the study since the research questions were observational.
Blinding	Blinding was not used because patient groups were defined by mutation statuses, which were not identified a priori (after sample collection and analysis design).

Reporting for specific materials, systems and methods

We require information from authors about some types of materials, experimental systems and methods used in many studies. Here, indicate whether each material, system or method listed is relevant to your study. If you are not sure if a list item applies to your research, read the appropriate section before selecting a response.

Materials & experimental systems

n/a	Involved in the study
☒	☐ Antibodies
☒	☐ Eukaryotic cell lines
☒	☐ Palaeontology and archaeology
☒	☐ Animals and other organisms
☐	☒ Human research participants
☐	☒ Clinical data
☒	☐ Dual use research of concern

Methods

n/a	Involved in the study
☒	☐ ChIP-seq
☒	☐ Flow cytometry
☒	☐ MRI-based neuroimaging

Human research participants

Policy information about [studies involving human research participants](#)

Population characteristics	The study cohort contained 32,442 patients who had a prior solid tumor diagnosis. The patients in this study had no record of hematologic malignancies within 3 months following sequencing. 55% of the cohort were female. The median age was 61. The cohort contained 2286 individuals with Asian ancestry, 2088 with Black ancestry, 24984 with White ancestry and 2738 with other reported ancestry. 15767 patients reported prior smoking history while 15103 did not. The median length of follow-up was 19.7 months.
Recruitment	We included any patient who underwent prospective sequencing (MSK-IMPACT) from 1/1/2014 to 2/1/2020 and who did not fulfill any of our defined exclusion criteria (described above). It is possible that patients who died in between initial presentation and IMPACT sequencing were censored leading to a left truncation of the survival data. However, the impact on our results is likely minimal considering our event of interest was hematological cancer diagnosis.
Ethics oversight	MSK-IMPACT panel was approved by an institutional prospective sequencing protocol (ClinicalTrials.gov number, NCT01775072). All patients enrolled on this protocol provided informed consent. The analysis plan, sample collection, and data usage were approved by the MSKCC Institutional Review Board (IRB). A subset of patients that underwent tumor-genomic profiling as standard of care were not directly consented, in which case an IRB waiver was obtained to allow for inclusion into this study.

Note that full information on the approval of the study protocol must also be provided in the manuscript.

Clinical data

Policy information about [clinical studies](#)

All manuscripts should comply with the ICMJE [guidelines for publication of clinical research](#) and a completed [CONSORT checklist](#) must be included with all submissions.

Clinical trial registration	NCT01775072
Study protocol	https://clinicaltrials.gov/ct2/show/NCT01775072
Data collection	We accessed sequence data of patients who underwent prospective sequencing (MSK-IMPACT) from 1/1/2014 to 2/1/2020 through an institutional, secure, PHI-free storage location.
Outcomes	For the development of hematologic neoplasms, the clinical endpoint was the time from blood draw to the development of a hematologic neoplasm, or censoring in cases lost to follow-up or who died. This was captured by medical codes (ICD-O, ICD-9/10) as well as manual review of patient electronic medical records. The study is observational and was not part of a clinical trial. No additional endpoints -- primary or secondary -- were considered outside of the development of a hematologic neoplasm.
